# Rapid Parametric Mapping of the Longitudinal Relaxation Time T_1_ Using Two-Dimensional Variable Flip Angle Magnetic Resonance Imaging at 1.5 Tesla, 3 Tesla, and 7 Tesla

**DOI:** 10.1371/journal.pone.0091318

**Published:** 2014-03-12

**Authors:** Matthias A. Dieringer, Michael Deimling, Davide Santoro, Jens Wuerfel, Vince I. Madai, Jan Sobesky, Florian von Knobelsdorff-Brenkenhoff, Jeanette Schulz-Menger, Thoralf Niendorf

**Affiliations:** 1 Berlin Ultrahigh Field Facility (B.U.F.F.), Max-Delbrueck Center for Molecular Medicine, Berlin, Germany; 2 Working Group on Cardiovascular Magnetic Resonance, Experimental and Clinical Research Center, a joint cooperation between the Charité Medical Faculty and the Max-Delbrueck Center for Molecular Medicine, HELIOS Clinics Berlin Buch, Department of Cardiology and Nephrology, Berlin, Germany; 3 Siemens Healthcare, Erlangen, Germany; 4 Institute of Neuroradiology, University Medicine Göttingen, Göttingen, Germany; 5 NeuroCure Clinical Research Center, Charité University Medicine Berlin, Berlin, Germany; 6 Clinic for Neurology & Center for Stroke Research Berlin, Charité Medical Faculty Berlin, Berlin, Germany; 7 Experimental and Clinical Research Center, a joint cooperation between the Charité Medical Faculty and the Max-Delbrueck Center for Molecular Medicine, Berlin, Germany; University of Maryland, College Park, United States of America

## Abstract

**Introduction:**

Visual but subjective reading of longitudinal relaxation time (T_1_) weighted magnetic resonance images is commonly used for the detection of brain pathologies. For this non-quantitative measure, diagnostic quality depends on hardware configuration, imaging parameters, radio frequency transmission field (*B_1_^+^*) uniformity, as well as observer experience. Parametric quantification of the tissue T_1_ relaxation parameter offsets the propensity for these effects, but is typically time consuming. For this reason, this study examines the feasibility of rapid 2D T_1_ quantification using a variable flip angles (VFA) approach at magnetic field strengths of 1.5 Tesla, 3 Tesla, and 7 Tesla. These efforts include validation in phantom experiments and application for brain T_1_ mapping.

**Methods:**

T_1_ quantification included simulations of the Bloch equations to correct for slice profile imperfections, and a correction for *B_1_^+^*. Fast gradient echo acquisitions were conducted using three adjusted flip angles for the proposed T_1_ quantification approach that was benchmarked against slice profile uncorrected 2D VFA and an inversion-recovery spin-echo based reference method. Brain T_1_ mapping was performed in six healthy subjects, one multiple sclerosis patient, and one stroke patient.

**Results:**

Phantom experiments showed a mean T_1_ estimation error of (-63±1.5)% for slice profile uncorrected 2D VFA and (0.2±1.4)% for the proposed approach compared to the reference method. Scan time for single slice T_1_ mapping including *B_1_^+^* mapping could be reduced to 5 seconds using an in-plane resolution of (2×2) mm^2^, which equals a scan time reduction of more than 99% compared to the reference method.

**Conclusion:**

Our results demonstrate that rapid 2D T_1_ quantification using a variable flip angle approach is feasible at 1.5T/3T/7T. It represents a valuable alternative for rapid T_1_ mapping due to the gain in speed versus conventional approaches. This progress may serve to enhance the capabilities of parametric MR based lesion detection and brain tissue characterization.

## Introduction

Magnetic resonance imaging (MRI) offers capabilities for non-invasive tissue characterization for a broad range of MRI applications including neuroinflammatory diseases and stroke [1], [2], [3], [4], [5]. At high and ultrahigh magnetic field strengths, brain parenchyma can be depicted with higher spatial resolution, which improves morphological conspicuity and facilitates improved characterization of multiple sclerosis (MS) plaques [6], [7], [8], enhances differential diagnosis of orphan neuorinflammatory diseases [9], [10], [11] and stroke [12]. Visual assessment of T_1_ weighted techniques [13], [14], [15] is a common standard in today's clinical brain imaging practice, although the diagnostic efficacy of cerebral lesion detection depends on the severity of focal or regional (patho-)physiological changes, on the imaging technique and pulse sequence design/timing used, on the impact of hardware configuration, as well as on the observer experience. Notwithstanding the ubiquity and success of clinical T_1_ weighted imaging, quantification of brain parenchymal T_1_ values is of great research interest and of substantial clinical relevance [16], [17], [18] but comes with the caveat of typically being rather time consuming.

T_1_ quantification techniques using variable flip angles (VFA), such as *driven equilibrium single pulse observation of T_1_* (DESPOT1, [19]) have been proposed for fast three dimensional T_1_ mapping of the brain. DESPOT1 includes at least two radio frequency (RF) spoiled gradient echo (FLASH [20]) measurements using different flip angles from which T_1_ maps with full brain coverage can be achieved in approximately 10 minutes scan time [21]. Not all clinical indications necessarily require whole brain coverage, but might rather benefit from fast 2D zonal imaging covering particular brain regions or lesions using a high in-plane spatial resolution together with a limited number of slices. Consequently, targeting rapid 2D mapping approaches is conceptually appealing for clinical T_1_ quantification of brain tissue. However, commonly used non-ideal radiofrequency (RF) pulses evoke an inhomogeneous slice excitation leading to deformed slice profiles [22]. Slice profile deformation presents an extra challenge for VFA based T_1_ mapping and bears the potential to deem T_1_ quantification inaccurate. Recognizing the constraints of conventional 2D VFA and the opportunities of 2D T_1_ mapping, this study examines the feasibility and fidelity of rapid slice profile corrected 2D VFA T_1_ mapping. To meet this goal, numerical simulations of the Bloch equations are used, which account for non-ideal RF pulse shapes and RF transmission field (*B_1_^+^*) non-uniformities. The applicability of this approach is demonstrated in phantom experiments at 1.5T, 3T and 7T. *In vivo* feasibility studies including healthy subjects, MS and stroke patients are conducted as a precursor to a broader clinical study. The merits and limitations of the proposed 2D VFA T_1_ mapping variant are discussed and implications for clinical imaging are considered.

## Methods

### Theory

Fast radio frequency (RF) spoiled gradient echo (FLASH) measurements using at least two flip angles allow for the quantification of the longitudinal relaxation time T_1_ [19]. The commonly accepted equation that governs FLASH signal intensity S(α) in the steady state is [23]

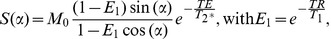
(Eq. 1)where M_0_ is the proton density, TR is the repetition time, TE is the echo time, α is the flip angle, and T_2_* is the effective transversal relaxation time. Rearrangement of equation 1 yields [24]

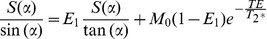
(Eq. 2)


Arranging S(α)/sin(α) over S(α)/tan(α) allows for extraction of T_1_ from a linear fit T_1_ = -TR/ln(m), where m is the slope between measurement points.

### Bloch equation simulations

Equation 1 and 2 hold true for excitations that exhibit a uniform flip angle over the entire target area. In 2D acquisitions, however, non-ideal RF pulse shapes lead to deformations of the slice profile altering the resulting signal intensities. To assess the extent of slice profile deformation and its impact on variable flip angle T_1_ quantification, a Matlab (TheMathworks, Natick, MA, USA) environment was developed allowing for simulations according to the Bloch equations and emulation of a FLASH sequence. A bandwidth truncated sinc RF pulse (bandwidth = 1 kHz, duration = 2 ms, time-bandwidth-product = 2) used for FLASH imaging was extracted from the sequence development environment of the “integrated development environment for (MR) applications” (IDEA, Siemens Healthcare, Erlangen, Germany) and fed into the simulation. One-dimensional magnetization vector profiles along the slice selection direction were generated in the steady state for given flip angles.

### T_1_ quantification: Simulated versus experimental signals

Using mathematical integration of the simulated slice profiles for different flip angles, theoretically achievable signal intensities were calculated. An iterative least squares minimization function matched these theoretical signal intensities to measured FLASH MR signal intensities by varying T_1_ between 1 ms and 10000 ms starting at T_1_ = 1000 ms and adapting the scaling factor M_0_. Pixel-by-pixel application of this method facilitated T_1_ mapping. The simulation environment allowed inclusion of afore acquired *B_1_^+^* data that were derived from RF transmission field mapping.

### Three flip angles approach

Further to the conventional approach that uses two flip angles we propose the application of three flip angles for VFA based T_1_ quantification. The first flip angle α_1_ was chosen to achieve the maximum signal intensity determined by Bloch simulations using the specific target T_1_. To determine the remaining two flip angles, α_2_ and α_3_ were varied from 1° to 90° including all possible combinations. Gaussian noise was added to the corresponding signal and T_1_ evaluations using the proposed approach were performed for all flip angle combinations. Flip angle sets resulting in minimal T_1_ differences between the theoretical T_1_ value and the estimated T_1_ value were regarded as adjusted flip angles and were henceforth used as nominal flip angles for the phantom experiments and for the *in vivo* studies.

In the first step, these one-time calculations were performed for T_1_ of a manganese chloride doped water phantom (T_1_ = 1000 ms) described below. In the second step, approximated mean T_1_ values found in the literature for gray and white matter at different field strengths (T_1_ = 950 ms at 1.5T [25], [26], [27], [28], T_1_ = 1250 ms at 3T [25], [27], [29], [30], [31], and T_1_ = 1650 ms at 7T [27]) were targeted.

### Monte Carlo simulations

To assess T_1_ dependent accuracy (defined as deviation of mean T_1_ value from nominal T_1_ value) and precision index (defined as standard deviation of T_1_ divided by mean T_1_) of the proposed mapping method and to compare the three flip angles approach with the two flip angles approach, Monte Carlo simulations of the proposed corrected 2D VFA fitting algorithm were performed for T_1_ values of 1 ms to 3000 ms using a 1 ms increment. Gaussian noise was added to the signal intensities so that a SNR margin of 100 between the highest of all three signals and the standard deviation of the noise was achieved. 10000 simulation experiments were conducted for each T_1_ using the adjusted flip angle set for the respective T_1_ values.

### RF transmission field (B_1_^+^) mapping

As VFA methods rely on a priori knowledge of the exact flip angle in the region of interest, correction for RF transmission field (*B_1_^+^*) non-uniformities is essential. *B_1_^+^* mapping was done using a Bloch-Siegert [32] implementation for phase based *B_1_^+^* mapping [33] using TR≈100 ms, Fermi pulse flip angle = 400° (nominal *B_1_^+^* = 4.35 μT), and a Fermi pulse off-center frequency = 4 kHz. Spatial resolution was adapted to the respective T_1_ mapping protocol. The sequence employed double gradient echo acquisitions with different echo times adapted to the corresponding fat/water frequency shift for each field strength to enable *B_0_*-mapping [34]. Calculation of transmission field distribution normalized to the nominal *B_1_^+^* was done offline using Bloch simulations in Matlab that also considered *B_0_* off-resonances.

### MR hardware

Measurements were performed on 1.5T/3T/7T whole body MRI systems (Avanto/Verio/Magnetom 7T, Siemens Healthcare, Erlangen, Germany) running identical software versions. At 1.5T and 3T a 12 channel (Siemens Healthcare, Erlangen, Germany) head coil was used for signal reception (Rx) while at 7T a 24 channel Rx coil (Nova Medical, Andover, MA, USA) was employed. For transmission (Tx), the integrated body coil was used at 1.5T and 3T while the head coil's Tx channel was used at 7T.

### Validation in Phantom Experiments

An oval brain like shaped water based phantom (diameter = 10 cm, length = 20 cm, T_1_ = 960 ms, T_2_ = 100 ms, measured at 1.5 T) was built to mimic mean gray and white matter T_1_ values at 1.5T. Manganese chloride was used to adjust T_1_, sodium chloride was used to adjust the conductivity to that of brain tissue [35].

To assess the capability of the Bloch simulations to accurately emulate slice excitation, the simulated magnetization vector profiles were compared to excitation profiles measured exemplarily at α = 30° at 3.0 T employing a modified FLASH sequence with the readout gradient along the slice selection direction.

Signal intensities over different flip angles calculated from Bloch simulations using the actual sequence configuration were compared to signal intensities derived from standard FLASH measurements (TE/TR = 2.5/5 ms; α = 2°−90°, voxel size = (2×2×5) mm^3^).

A two-dimensional inversion recovery (IR) prepared technique (voxel size: (2×2×5) mm^3^, non-selective adiabatic hyperbolic secant inversion, echo spacing = 5.5 ms, TR = 10 s, α = 90°, turbo factor = 5, GRAPPA acceleration factor = 2, 8 inversion times of TI = 60/120/240/480/750/1000/3000/5000 ms, scan time = 16 min) in conjunction with a spin-echo readout served as a reference for T_1_ quantification. T_1_ was calculated offline in Matlab using a non-linear least squares three-parameter fit.

To validate the feasibility and accuracy of the corrected 2D VFA T_1_ mapping method accounting for slice profile deformations, 2D FLASH images (TE/TR = 2.5/5 ms, scan time = 3.5 s) using a set of three adjusted flip angles were acquired. *B_1_^+^*-maps were used to account for *B_1_^+^* non-uniformities.

T_1_ of the phantom was quantified for all field strengths (1.5T, 3T, and 7T). For this purpose, a region of interest covering the entire cross-section of a central coronal slice of the phantom was used. Mean T_1_ values and standard deviations were calculated for this ROI. To elucidate the impact of the signal change due to slice profile deformation on the 2D quantification of T_1_, conventional *B_1_^+^*-corrected VFA T_1_ evaluation used for 3D acquisitions that did not consider slice profiles were performed using two flip angles (2°/13°) suggested by the 3D approach [36]. For reasons of brevity, the expression “uncorrected 2D VFA” is used for this approach in the following. The proposed 2D *B_1_^+^* corrected VFA T_1_ mapping approach that includes extra slice profile deformation versus the conventional approach is called “corrected 2D VFA”.

### Ethics Statement

For the *in vivo* feasibility study, 6 healthy subjects (mean age: 28±2 years, 2 females) without any known history of brain disease, one MS patient (age: 46 years, female), and one stroke patient (24 years, male) underwent MR imaging after due approval by the ethical committees (registration number DE/CA73/5550/09, Landesamt für Arbeitsschutz, Gesundheitsschutz und technische Sicherheit, Berlin, Germany and registration number DRKS00003193, 7 Tesla Ultra-High Field Project 7UP, WHO International). Informed written consent was obtained from each volunteer and patient prior to the study.

### *In vivo* studies in healthy subjects and patients

Axial 2D brain images were obtained (FLASH, TE/TR = 2.5 ms/5 ms, scan time = 3.5 s) using flip angle triples, which were adjusted to mitigate saturation and noise induced errors. Gray matter (GM) and white matter (WM) were segmented by thresholding a combination of T_1_ weighted images and T_1_ maps derived from the reference T_1_ measurement. The resulting gray matter and white matter masks were applied to the uncorrected 2D VFA approach and to the proposed corrected 2D VFA. The IR-SE based T_1_ quantification reference method described for the phantom measurements was also used for the volunteer study. Mean T_1_ and standard deviations were calculated for the reference method and for slice profile deformation corrected 2D VFA. For volunteer studies, a spatial resolution of (2×2×5) mm^3^ was used for data acquisition, which was interpolated to (1×1×5) mm^3^ during reconstruction to improve visual appearance. For patient studies, the spatial resolution was adjusted to (1.6×1.6×4) mm^3^ during the acquisitions and interpolated to (0.8×0.8×4) mm^3^ in the reconstruction.

## Results

### Determination of adjusted flip angles using Bloch simulations

Optimum flip angles for T_1_ quantification of the phantom were calculated to be 3°/11°/30° at 1.5T, 3°/11°/28° at 3T, and 3°/11°/26° at 7T. Adjusted flip angles for T_1_ quantification *in vivo* were found to be 3°/11°/26° at 1.5T, 3°/9°/22° at 3T, and 3°/8°/25° at 7T.

### Monte Carlo simulations

**Figure 1:**
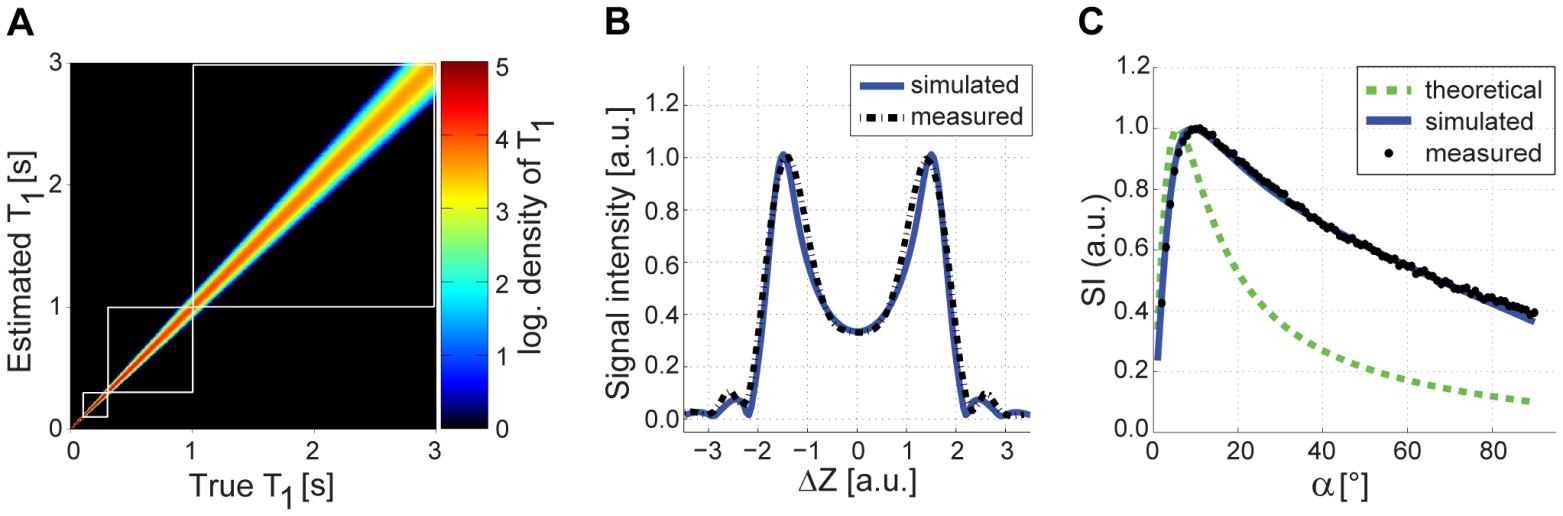
Monte Carlo simulation and impact of slice profile deformation on FLASH signal. (**a**) Simulated density distribution (logarithmic) of true T_1_ values versus estimated T_1_ values derived from corrected 2D VFA using three flip angles. For each T_1_ value, 10000 experiments were performed. The use of three flip angles improved the T_1_ precision index by 11% compared using only two flip angles. (**b**) Bloch simulated (blue solid line) and measured (black dashed line) slice profiles (signal relevant component) in the steady state of a FLASH sequence show good agreement. The slice profile substantially deviates from an ideal rectangular slice profile. (**c**) Theoretical (green dashed line), Bloch simulated (blue solid line), and measured (black dots) signal intensities for FLASH as a function of the flip angle (at T_1_ = 1000 ms). The Bloch simulated signal intensities agree very well with the phantom measurements.

Figure 1a shows results derived from Monte Carlo simulation of the proposed corrected 2D VFA approach for T_1_ relaxation times ranging from 0–3 s. T_1_ precision decreases with increasing T_1_, while accuracy remains unaffected. If a two flip angle approach is used as a reference, the T_1_ precision index was improved for the three flip angles approach by 9% for T_1_ values ranging from 100 ms to 300 ms, by 13% for T_1_ values ranging from 300 ms to 1000 ms, and by 12% for T_1_ values ranging from 1000 ms to 3000 ms.

### Validation in phantom experiments

Bloch simulated and measured excitation slice profiles derived from the phantom are depicted in Figure 1b for an excitation pulse of α = 30°. Simulation and measurement agree very well. The saturation dependent slice profile deformation is characterized by the RF pulse shape, TR, T_1_ and the flip angle. The slice profile deformation and *B_1_^+^* non-uniformity induced discrepancy between theoretical and measured signal curves for FLASH is demonstrated in Figure 1c. While theoretical signal intensities using Eq. 2 deviated substantially from the measurements, the signal curve derived from the *B_1_^+^* corrected simulations including slice profile considerations perfectly matched the experimental data. The flip angle providing maximum signal was found to be 11° (considering slice profile deformations), which is almost twice the theoretical Ernst angle of 6°.

**Figure 2:**
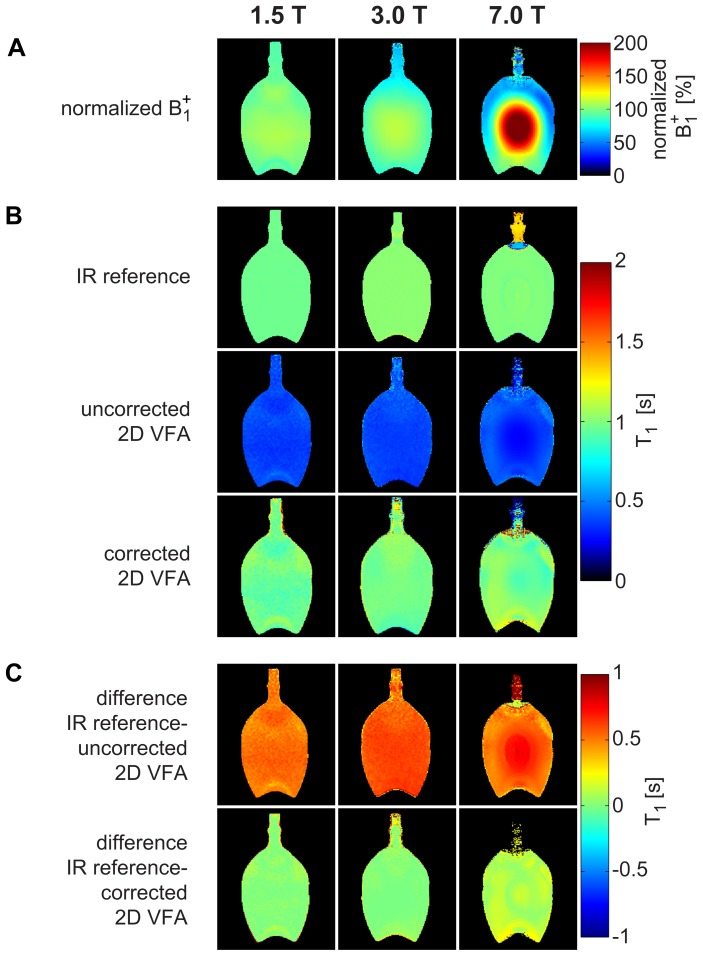
Normalized *B_1_^+^* maps and T_1_ maps of the water phantom at 1.5T, 3T, and 7T. (**a**) Measured *B_1_^+^* normalized to the nominal *B_1_^+^* (4,35 μT) in percent. (**b, top row**) T_1_ derived from the inversion recovery reference method (T_1_ = (963±7)ms at 1.5T, T_1_ = (1022±10)ms at 3T, T_1_ = (1002±10)ms at 7T). (**b, center row**) Uncorrected 2D VFA approach that does only consider *B_1_^+^* but not slice profile deformations. Adjusted flip angles (α_1_ = 2°, α_2_ = 13°) were used. This approach revealed a T_1_ underestimation of −63.0±1.5% averaged over all field strengths (T_1_ = (372±28)ms at 1.5T, T_1_ = (376±23)ms at 3T, T_1_ = (357±60)ms at 7T) compared to the reference measurement. (**b, bottom row**) With the proposed *B_1_^+^* corrected and slice profile distortion corrected 2D VFA measured a T_1_ deviation of only 0.2±1.4% averaged over all field strengths (T_1_ = (966±67)ms at 1.5T, T_1_ = (1010±47)ms at 3T, T_1_ = (1017±89)ms at 7T) compared to the reference measurement. (**c, top row**) Difference map between uncorrected 2D VFA and IR reference. (**c, bottom row**) Difference map between corrected 2D VFA and IR reference.

*B_1_^+^* maps of the phantom at 1.5T, 3T, and 7T are shown in Figure 2a. As expected, the *B_1_^+^* homogeneity decreases with increasing field strength. At 1.5T, T_1_ quantification using the inversion recovery approach as a reference yielded T_1_ = (963±7)ms for the cross-sectional ROI of the phantom. At 3T, the reference T_1_ value was found to be (1022±10)ms. For the same phantom, T_1_ = (1002±10)ms was obtained at 7T as illustrated in Figure 2b. Conventional, only *B_1_^+^* corrected 2D VFA T_1_ mapping using Eq. 2 substantially underestimated T_1_ and yielded T_1_ = (372±28)ms at 1.5T, T_1_ = (376±23)ms at 3T, and T_1_ = (357±60)ms at 7T as demonstrated in Figure 2b. The inhomogeneous T_1_ map at 7T also underlines that a solitary *B_1_^+^* correction is not sufficient to compensate for the non-linear flip angle dependent signal changes. In comparison, slice profile deformation and *B_1_^+^* corrected 2D VFA provided T_1_ = (966±67)ms at 1.5T, T_1_ = (1010±47)ms at 3T and T_1_ = (1017±89)ms at 7T as illustrated in Figure 2b, which matches the reference data fairly well. The proposed slice profile corrected 2D VFA approach yielded a mean deviation of T_1_ from the reference value as low as (0.2±1.4)% averaged over all field strengths (Figure 2c). In comparison, an error in T_1_ quantification of (−63±1.5)% was observed for the 2D VFA technique without slice profile deformation correction as shown in Figure 2c.

All measurements, including T_1_ reference measurements, showed inconsistent results in the neck of the water bottle at 7T, because this region was outside of the covered volume of the local transmission coil.

### Volunteer and Patient Studies

**Figure 3:**
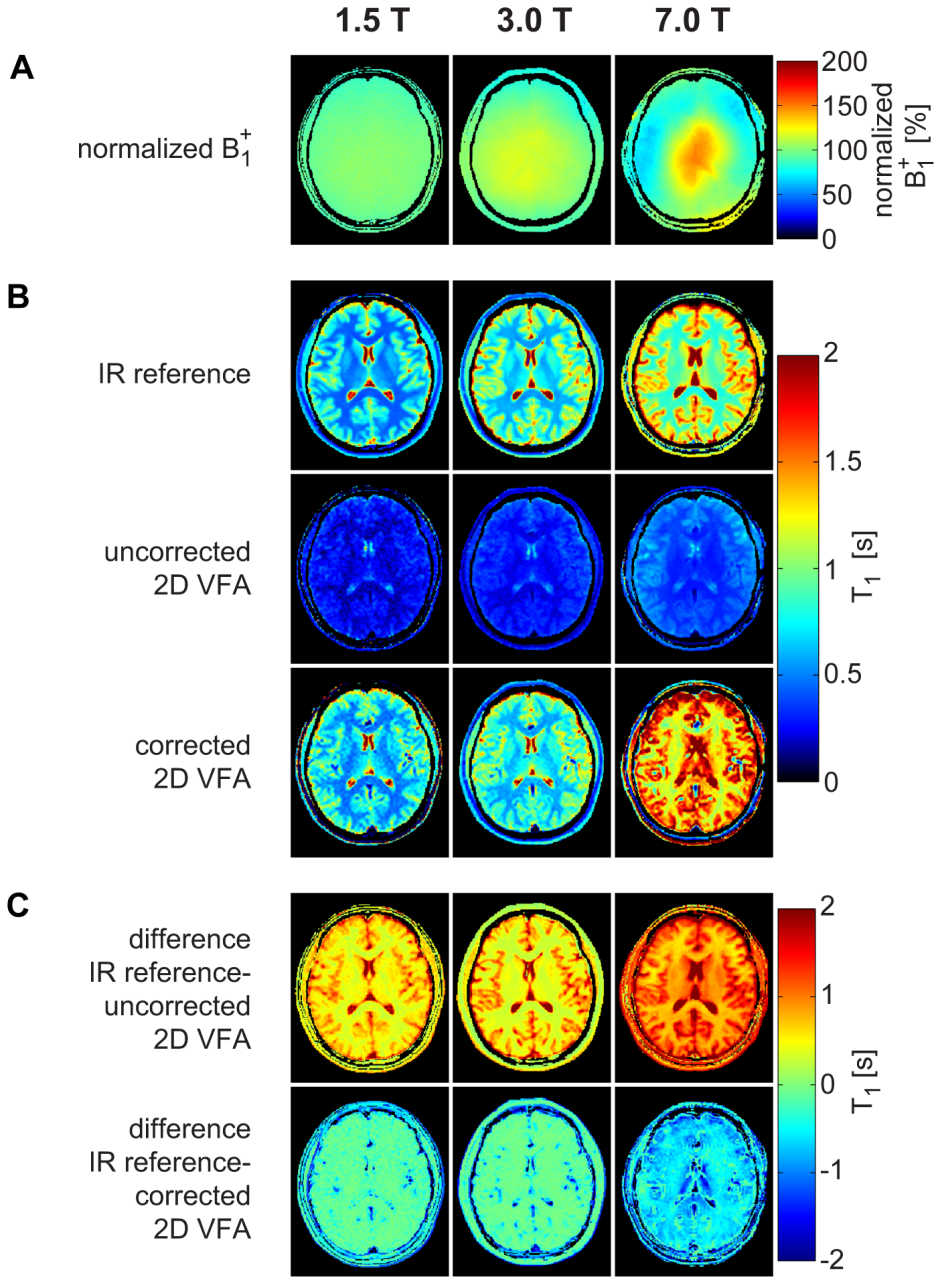
*B_1_^+^* mapping and T_1_ mapping in healthy volunteers at 1.5T, 3T, and 7T. Exemplary normalized *B_1_^+^* maps (**a**) and T_1_ maps (**b**) of a healthy brain using the inversion recovery reference measurement, the uncorrected 2D VFA, and the proposed corrected 2D VFA approach at 1.5T, 3T, and 7T. The difference maps between IR reference and uncorrected/corrected 2D VFA are illustrated in (**c**).

T_1_ mapping performed in healthy volunteers at 1.5T, 3T, and 7T using the IR-SE reference measurement, the uncorrected 2D VFA, and the corrected 2D VFA are depicted in Figure 3. T_1_ values for gray matter and white matter are illustrated in Table 1.

**Table 1:** T_1_ values for gray matter and white matter in healthy volunteers at 1.5T, 3T, and 7T derived from IR-SE reference measurement, uncorrected 2D VFA, and corrected 2D VFA.

		T_1_ [ms]		
		1.5T	3T	7T
**white matter**	IR reference	678±10	911±15	1284±22
	uncorrected 2D VFA	298±14	377±25	554±21
	corrected 2D VFA	791±21	969±85	1855±141
**gray matter**	IR reference	1154±82	1615±149	2065±69
	uncorrected 2D VFA	465±40	518±42	804±48
	corrected 2D VFA	1282±78	1433±80	2524±137

At 1.5T, flip angles were 3°/11°/26° for the proposed corrected 2D VFA and 2°/13° for uncorrected 2D VFA. The absolute mean difference between IR reference and uncorrected 2D VFA was 60% for gray matter and 56% for white matter. The absolute mean difference between IR reference and corrected 2D VFA was 11% for gray matter and 17% for white matter.

At 3T, flip angles were 2°/12° for uncorrected 2D VFA and 3°/9°/22° for the proposed corrected 2D VFA. The absolute mean difference between IR reference and uncorrected 2D VFA was 68% for gray matter and 59% for white matter. The absolute mean difference between IR reference and corrected 2D VFA was 11% for gray matter and 6% for white matter.

At 7T, flip angles were 2°/11° for uncorrected 2D VFA and 3°/8°/25° for the proposed corrected 2D VFA. The absolute mean difference between IR reference and uncorrected 2D VFA was 61% for gray matter and 57% for white matter. The absolute mean difference between IR reference and corrected 2D VFA was 22% for gray matter and 44% for white matter.

The acquisition time for the IR-SE reference measurement was 16 minutes for each slice at each field strength. For corrected 2D VFA T_1_ mapping, acquisition time including *B_1_^+^* mapping was 18 s for each slice.

**Figure 4:**
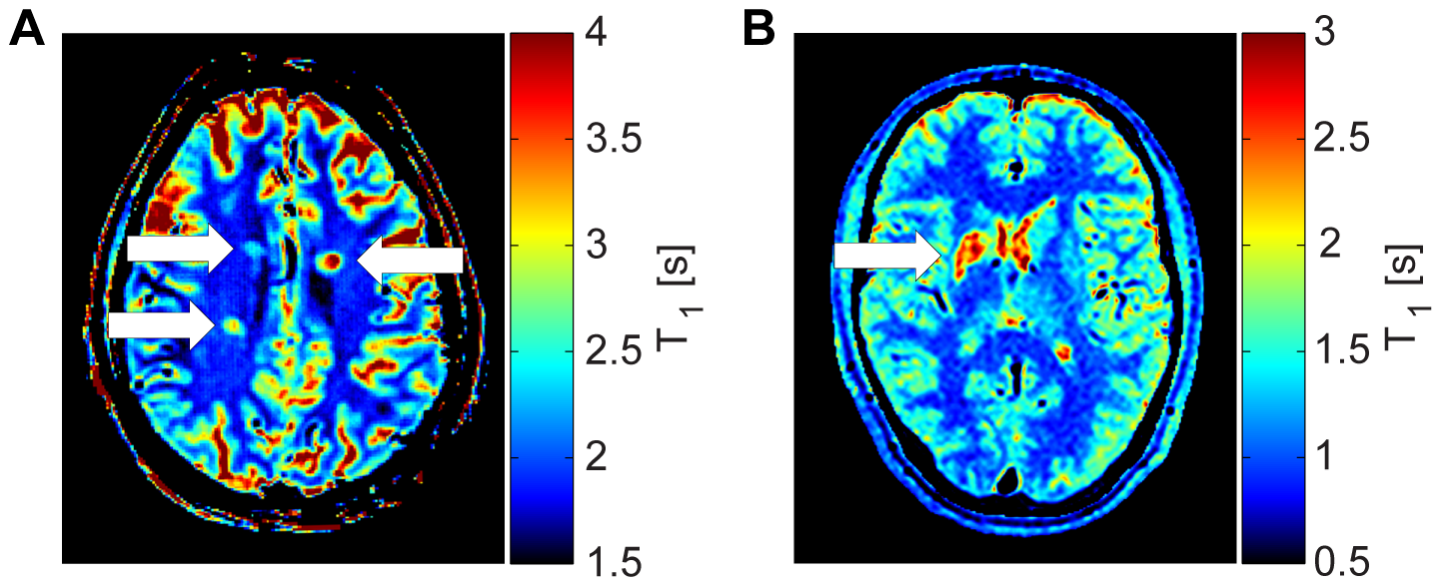
Patient measurements using the corrected 2D VFA method. (**a**) T_1_-map of a multiple sclerosis patient at 7T using the corrected 2D VFA method. The map shows three periventricular lesions (white arrows). Mean T_1_ of the lesions was (2758±615)ms vs. (1842±62)ms in the surrounding white matter. (**b**) Corrected 2D VFA T_1_-map of a stroke patient derived from 3T acquisitions showing a subcortical lesion (white arrow). The stroke occurred five months before measurement. T_1_ of the lesion was (2554±316)ms vs. (951±74)ms in the surrounding white matter.

The T_1_ measurement of the MS patient at 7T revealed (2758±615)ms in three periventricular lesions vs. (1842±62)ms in the surrounding white matter (Fig. 4a). T_1_ in the subcortical lesion of the stroke patient was (2554±316)ms vs. (951±74)ms of the surrounding white matter at 3T (Fig. 4b).

## Discussion

This study demonstrates the feasibility of rapid 2D T_1_ quantification using variable flip angles in phantom experiments and *in vivo* at magnetic field strengths of 1.5T, 3T and 7T. For this purpose, implications of RF transmission field non-uniformities, as well as excitation slice profile deformations due to non-ideal RF pulses were carefully considered. For correction of slice profile deformation induced errors, Bloch simulations were employed. Corrected 2D VFA T_1_ mapping was found to be accurate when compared to reference inversion recovery acquisitions in phantom studies at all tested field strengths. For T_1_ mapping of the brain, mean T_1_ values measured by corrected 2D VFA show a fairly good agreement with the reference measurement at 1.5T and 3T for both gray and white matter. T_1_ relaxation times found for corrected 2D VFA at 7T were larger than that derived from IR-SE. The applicability and efficacy of the proposed procedure to calculate adjusted flip angles in order to mitigate noise induced errors was confirmed by our simulations and experimental data.

The inclusion of *B_1_^+^* maps made 2D VFA T_1_ mapping immune to non-uniformities in the *B_1_^+^* transmission field, which are pronounced at high and ultrahigh field strengths. Magnitude based *B_1_^+^* mapping methods such as double angle [37], [38], [39], [40] or the use of inversion recovery prepared extra scans [21], [41] have been presented previously to correct for flip angle inaccuracies. However, most of these techniques rely on equilibrium magnetization before each acquisition or need extra calibration routines and therefore add extra scan time. Flash-EPI hybrid sequences employing adiabatic pulses [42] were proposed to potentially provide means for overcoming *B_1_^+^* inhomogeneities while maintaining reasonable scan time. However, SAR constraints dictate flip angle limits and adiabatic pulses demand longer echo times especially at higher magnetic fields, which lead to partial dephasing of the magnetization. The phase based *B_1_^+^* mapping technique used here is fast and reliable, even in low signal regions [33] of the brain. The rapid data acquisition reduces motion sensitivity in *B_1_^+^* mapping, which is ultimately beneficial for in vivo T_1_ quantification.

The reference method was already accelerated by using parallel imaging with an effective reduction factor of R = 2 together with a spin echo turbo factor of 5 and a reduced phase field of view. The corrected 2D VFA implementation used for the volunteers offers a scan time advantage factor of approximately 570 versus the unaccelerated inversion recovery reference method. Obviously, corrected 2D VFA supports modest accelerations, which would further enhance the speed advantage over the reference. Notwithstanding the utility of the reference method that relied on equilibrium magnetization before each inversion pulse for T_1_ quantification, the potential to shorten scan times is limited. As T_1_ of brain tissue increases with field strength, the equilibrium condition of TR>5×T_1_ requires even longer TRs, which further increase scan time. Unlike the reference method, moving to higher magnetic fields does not add a scan time penalty to corrected 2D VFA. Even more, the lack of inversion pulses in 2D VFA reduces magnetization transfer effects and RF power deposition.

Our results demonstrate severe T_1_ quantification errors due to slice profile deformations in the uncorrected 2D VFA T_1_ mapping using commonly accepted signal equations. This underscores that theoretical signal predictions only hold true for ideal rectangular slice profiles assuming uniform excitation over the entire slice or volume. Slice selection RF pulses are however bandwidth limited and lead to a spatially non-uniform flip angle distribution in slice selection direction. This evokes T_1_-, TR-, and flip angle dependent saturation phenomena that create distorted slice profiles. Consequently, it is essential to correct for slice deformations before T_1_ values derived from 2D VFA acquisitions can be considered accurate.

The match of T_1_ values derived from IR-SE reference measurements with T_1_ values derived from the corrected 2D VFA at all tested field strengths suggests that the sequence related deviations were successfully addressed by our 2D VFA implementation. In-vivo T_1_ maps obtained with the two methods, however, revealed some residual differences. This underscores the challenges of parametric mapping when moving from phantoms to *in vivo*, where tissue characteristics become relevant and play a role for T_1_ quantification. It is to be expected that magnetization transfer weighting, weighting of T_1_ relaxation components, and T_2_ relaxation components in mixed tissue is different for different T_1_ mapping techniques, which ultimately leads to different mean T_1_ values. Therefore, it is challenging to establish normal values for tissue that are valid across all T_1_ quantification techniques. The broad variety of reference T_1_ values found in the literature supports this statement [25], [26], [27], [28], [29], [30], [31]. We also performed simulations using a three pool model of mixed white matter tissue (myelin, myelinated axons, and mixed water pools) together with volume fractions reported for adults [43]. The difference in mean T_1_ between the IR-SE reference and the proposed corrected 2D VFA was found to be 15%. In our measurements, the linear trend of increasing T_1_ with increasing field strength could not be shown for the corrected 2D VFA method at 7T. However, for all field strength the discrepancy between the reference measurement and corrected 2D VFA T_1_ mapping (mean deviation of 18%) was reduced versus the discrepancy between the reference and uncorrected 2D VFA T_1_ mapping (mean deviation of 63%).

Admittedly, it may take several excitations before the magnetization reaches steady state in gradient echo techniques, which depends on the RF pulse shape, T_1_ relaxation time, TR, and the flip angle used. For this reason, we integrated 200 dummy pulses for T_1_ mapping to approximate steady state. This approach resulted in total scan times of approximately 18 s for single slice 2D VFA T_1_ mapping in the phantom and in healthy volunteers (spatial resolution = 2×2×5 mm^3^) including all three flip angles and including *B_1_^+^* mapping. For the patient measurements, a clinically acceptable scan time of 30 s was achieved for single slice T_1_ mapping (spatial resolution = 1.6×1.6×4 mm^3^). As all variable flip angle T_1_ quantification techniques rely on the steady state approximation, fast moving blood might not allow the steady state to form, alter the signal, and thus bear the potential to deem blood T_1_ quantification inaccurate.

In order to maintain consistent scan protocols throughout all field strengths, we used an echo time of 2.5 ms and a repetition time of 5 ms due to specific absorption rate (SAR) considerations at 7T. It is a recognized limitation that this approach is suboptimal at 1.5T and 3T. Here, shortening of TR would further reduce scan time. Moreover, RF Pulse shapes, gradient strengths and slew rates, receiver bandwidth, and spatial resolution can be adapted to use the full range of the system's specification at 1.5T, 3T and 7T. The repetition times and scan times reported here for *B_0_* and *B_1_^+^* mapping were limited by SAR constraints at 7T and of course can be further shortened at 1.5T and 3T. Implementation of these changes affords T_1_ mapping for a single slice in 5 s including *B_1_^+^* mapping. To reduce the computational effort, an accelerated algorithm for T_1_ quantification was implemented in Matlab, which enables generation of T_1_ maps using a matrix size of 256×256 pixels in less than three seconds.

The proposed approach for the calculation of adjusted flip angles to reduce T_1_ quantification errors in 2D VFA T_1_ mapping by balancing dynamic range and SNR does not yet account for *B_1_^+^* inhomogeneities or RF transmitter mis-calibrations, which can be hardware-, setup-, subject-, and also organ specific. Therefore, the proposed approach requires proper RF transmitter adjustment – a procedure which is included in the pre-scan calibration of clinical scanners - in order to get as close as possible to the adjusted flip angles. The choice of flip angles depends on the grade of slice profile deformation and therefore varies with the RF pulse shape. Our simulations and experiments focused on a bandwidth truncated sinc RF pulse with a bandwidth of 1 kHz and a duration of 2 ms. For other RF excitation pulses, adjusted flip angles might be different and require a onetime pre-calculation before used for T_1_ mapping. In contrast to the theoretical assumptions based on signal equations, the deformation of the slice profile in 2D gradient echo acquisitions shifts the suggested flip angles to larger values. Consequently, these flip angles may be limited by SAR constraints, especially at high and ultrahigh field strengths. Bandwidth reduced RF pulses allow higher flip angles, however at the cost of a non-uniform slice excitation. Notwithstanding the possible limitations discussed here, the proposed 2D VFA T_1_ mapping approach is capable to deal with and correct for slice profile distortions and showed reliability even at high field strengths. The single slice approach presented in this work can be extended to multi-slice T_1_ mapping. As the effective slice thickness increases with increasing slice profile distortion, cross-talk between slices has to be mitigated by application of interleaved data acquisition or a sufficient inter-slice distance.

SNR constraints of 2D VFA T_1_ mapping remain a concern for tissue characterization using parametric mapping of relaxation times. Here our *B_1_^+^* corrected approach is promising, since it was found to support the use of many-element surface receiver coils that can provide SNR improvements and speed gain [44], [45].

## Conclusion

Corrected 2D VFA T_1_ mapping represents a valuable alternative for rapid T_1_ mapping due to the speed gain versus conventional approaches. This progress may serve to enhance the capabilities of parametric MR based lesion detection and brain tissue characterization. The benefits of such improvements would be in positive alignment with the needs of explorations that are designed to examine the potential of high and ultrahigh field MRI for the assessment of neurodegenerative and neuroinflammatory diseases including differential diagnosis of orphan diseases.
